# Combined translabyrinthine, anterior petrosal approach for resection of collision vestibular schwannoma and petrous apex meningioma in neurofibromatosis type 2, with auditory brainstem implant placement

**DOI:** 10.3171/2021.7.FOCVID21130

**Published:** 2021-10-01

**Authors:** Usman A. Khan, Jillian H. Plonsker, Rick A. Friedman, Marc S. Schwartz

**Affiliations:** 1 Department of Neurosurgery and; 2 Division of Head and Neck Surgery, University of California San Diego, California

**Keywords:** neurofibromatosis type 2, vestibular schwannoma, translabyrinthine, anterior petrosectomy, Kawase

## Abstract

The natural history of neurofibromatosis type 2 (NF2) is profound bilateral hearing loss. The decision to pursue microsurgery may be more complicated in NF2 than with sporadic tumors. Schwannomas in NF2 often occur with other skull base tumors. Treatment should be tailored to preserve auditory perception for as long as possible. The authors present the case of a man with NF2 and a vestibular schwannoma who has poor hearing on the same side as a large petrous apex meningioma, both opposite to a well-hearing ear. This case highlights surgical decision-making and technical nuances during resection of collision tumors in NF2.

The video can be found here: https://stream.cadmore.media/r10.3171/2021.7.FOCVID21130

## Transcript

This video demonstrates a combined right translabyrinthine and anterior petrosal approach for resection of a petrous apex meningioma in collision with a vestibular schwannoma in a young male with NF2.

### 0:34 Clinical Presentation

The patient is a 44-year-old with a history of shunting in childhood. He had several years of bilateral sensorineuronal hearing loss and debilitating exertional headaches, suggesting an acquired Chiari malformation. He has a history of unilateral vestibular schwannoma in his father. His neurological examination showed mild papilledema but was otherwise normal. An audiogram was performed, showing sensorineuronal hearing loss bilaterally with worse PTA and speech discrimination on the right.

### 1:09 Imaging

Preoperative MRI shows a large petrous apex meningioma with a dural base lateral to Meckel’s cave. This meningioma has a component that enters the posterior fossa to the level of the IAC (internal auditory canal), where enhancement shows a 2.5 × 2–cm vestibular schwannoma. Red circles highlight a contralateral 2.0 × 1–cm schwannoma, the hallmark of NF2.

### 1:37 Approach

This case highlights several management principles in NF2. This is a patient with a new diagnosis of NF2 with likely inheritance from his father, who carried either a very mild germline mutation or a mosaic form, which has been inherited as germline in the patient. This patient’s most pressing issue is the petrous apex meningioma. In our practice, recent worsening of Chiari headaches is a good indicator of tumor growth, and resection should be pursued.

**2:08** The natural history of NF2 is profound bilateral hearing loss, and surgery should be tailored to preserve hearing as long as possible. This patient had poor hearing on the right side and good hearing on the left, which made the recommendation to pursue resection of the right schwannoma straightforward. Resection of NF2 schwannoma in a good- or only-hearing ear makes this recommendation more challenging.

**2:33** Schwannomas in NF2 are often polyclonal, making functional and anatomical preservation of the cochlear nerve unfeasible. In this case, we routinely use auditory brainstem implants to restore auditory perception when the likelihood of durable hearing preservation in the contralateral ear is poor. This is typically done in the same sitting.

**2:59** With this in mind, we planned for a combined petrosal approach for resection. Meningioma resection is carried out after skull base exposure and aided by division of the tentorium. Resection of the vestibular schwannoma is then performed, followed by ABI.

**3:03** We begin with the translabyrinthine approach. A cortical mastoidectomy is performed. Here the mastoid antrum and the tegmen are identified, followed by the presigmoid and postsigmoid dura. This patient had a poorly pneumatized temporal bone.

**3:23** A diagram depicts the course of the facial nerve as well as the structures of the inner and middle ear. The labyrinth is opened, and the IAC is skeletonized, including the inferior and superior troughs. These should be carried as medially as possible to provide 270° of exposure around the IAC and tumor.

**3:59** With the translabyrinthine approach complete, we turn our attention to the middle fossa approach, beginning with peeling of the middle fossa dura from the posterior-to-anterior direction to protect greater superficial petrosal nerve. The diagram here depicts Kawase’s bone as well as its margins around the GSPN (greater superficial petrosal nerve) and the IAC. The IAC is first decompressed with a diamond bit. A monopolar stimulator can be used to define the GSPN laterally, and Kawase’s bone is drilled away with a diamond bit.

**4:49** We perform our resections without retractors whenever possible, and this is aided by early CSF (cerebrospinal fluid) release. In combined petrosal craniotomies, this should be done superior to the IAC tumor. Alternating internal debulking and extracapsular dissection helps define the meningioma in the posterior fossa. Fascicles of the fifth cranial nerve can be seen here alongside the pons.

**5:39** Further resection is possible through division of the tentorium.

**5:58** Here tumor is resected along the pons and where the meningioma enters Meckel’s cave. This is resected, and any remnant is cauterized.

**6:14** With meningioma resection complete, the dura of Trautman’s triangle is opened for resection of the vestibular schwannoma. The arachnoid plane between the tumor and the posterior fossa is developed with the monopolar stimulator, which allows for continuous monitoring of facial nerve EMG during the resection.

**6:42** Alternating extracapsular dissection and internal debulking allow for identification of the cochlear nerve, which should be divided sharply against the brainstem before cautery against the deeper parts of the tumor should be performed.

**7:01** In this case, the root entry zone of the facial nerve and the tumor had a poor plane, and we turned our attention to the lateral parts of the tumor at the fundus, where the plane between the tumor and facial nerve was better identified.

**7:47** The cochlear nerve is dissected alongside specs of tumor in the IAC, which can be lifted away with a nerve hook. Here is division of the cochlear nerve, followed by sharp dissection along the facial nerve, which allowed for identification of a remnant of the meningioma not seen in Kawase’s exposure.

**8:29** The last bits of the vestibular schwannoma are then resected using sharp dissection with microscissors in a lateral-to-medial direction.

**9:16** We achieved a gross-total resection of both the meningioma and vestibular schwannoma, and attention then turned to placement of the ABI. PICA and the choroid of the fourth ventricle can be seen here. The rhomboid lip of the fourth ventricle is identified; the ABI is prepared and inserted along the cochlear nucleus anterior and superior. This is supported with Teflon and fat. Fat is packed into the translabyrinthine approach and the wound is closed in layers.

### 9:52 Postoperative Course

The patient awoke with a House-Brackmann grade IV facial palsy, which had improved to grade III at early follow-up. A CT is shown here demonstrating bone removal from the translabyrinthine and anterior petrosectomy approaches. The patient had refused MRI while inpatient and is awaiting an intubated examination as an outpatient.

### 10:17 References^1–5^

## Disclosures

The authors report no conflict of interest concerning the materials or methods used in this study or the findings specified in this publication.

## Author Contributions

Primary surgeon: Schwartz, Friedman. Assistant surgeon: Khan. Editing and drafting the video and abstract: Khan. Critically revising the work: Schwartz, Khan. Reviewed submitted version of the work: Schwartz, Khan, Plonsker. Approved the final version of the work on behalf of all authors: Schwartz. Supervision: Schwartz.
